# What kind of patient benefits the most from an intensive physiotherapy after total hip replacement?

**DOI:** 10.1093/eurpub/ckac131.303

**Published:** 2022-10-25

**Authors:** L Dionisi, I Accame, F Praino, G Abinova, I Sanguineti, AM Gentile, N Nante

**Affiliations:** 1 Post Graduate School of Public Health, University of Siena, Siena, Italy; 2 San Michele, Private Hospital, Albenga, Italy; 3 Department of Molecular and Developmental Medicine, University of Siena, Siena, Italy

## Abstract

**Background:**

Hip replacement is a common orthopaedic surgery procedure that produces great improvement in the quality of life. Despite this, a globally standardized post-operative physiotherapy protocol still does not exist. The aim of this study is to identify the factors that influence the motor and functional outcome after early, intensive, hospitalized treatment.

**Methods:**

A retrospective study was conducted in 2019 on 509 patients admitted to an Italian private clinic specialized in post-surgery rehabilitation, which applies an original bio-psycho-social-environmental protocol and individual rehabilitation plans. Data regarding each patient were collected from medical records: age, haemoglobin, Body Mass Index (BMI), Cumulative Illness Rating Scale (CIRS), Tinetti scale (TS) and Barthel scale (BS) at admission and discharge. The outcome was measured as the difference (Δ) between the values at discharge and admission of BS (ΔBS) and TS (ΔTS). We performed a univariate linear regression using STATA, to determine which factors influence the outcome. A p < 0.05 was considered statistically significant.

**Results:**

Our sample (57.4% female) was 70.1 ± 10.4 years old. ΔBS was significantly influenced by motor performance at admission (BS: Coef. - 0.708, CI 95% [- 0.743 - - 0.673]; TS: Coef. - 1.697, CI 95% [- 1.849 - - 1.544]), global health conditions (CIRS Severity index: Coef. 4.925, CI 95% [1.037 - 8.814]) and age (Coef. 0.312, CI 95% [0.221 - 0,403]). ΔTS was significantly influenced by the same factors, BS: Coef. - 0.167, CI 95% [- 0.184 - - 0.149]; TS: Coef. - 0.667, CI 95% [- 0.703 - - 0.631]; CIRS Severity index: Coef. 1.254, CI 95% [0.012 - 2.511]; age: Coef. 0.090, CI 95% [0.061 - 0.120]. BMI and haemoglobin did not influence the studied outcomes.

**Conclusions:**

Patients with worse health conditions, advanced age and lower motor performance at admission obtain a higher outcome.

**Key messages:**

• A broader knowledge of rehabilitation influencing factors would provide higher outcomes.

• The diffusion of individual rehabilitation plans would allow the achievement of better results.

